# Role of circulating sphingolipids in lipid metabolism: Why dietary lipids matter

**DOI:** 10.3389/fnut.2022.1108098

**Published:** 2023-01-11

**Authors:** Catherine Calzada, Cécile Vors, Armelle Penhoat, David Cheillan, Marie-Caroline Michalski

**Affiliations:** ^1^Univ-Lyon, CarMeN Laboratory, Inserm U1060, INRAE UMR1397, Université Claude Bernard Lyon 1, Pierre Bénite, France; ^2^Service de Biochimie et de Biologie Moléculaire, Centre de Biologie et de Pathologie Est, Hospices Civils de Lyon, Bron, France

**Keywords:** sphingolipids, ceramide, sphingomyelin, dietary lipids, nutrition, postprandial metabolism

## Abstract

Sphingolipids are structural components of cell membranes and lipoproteins but also act as signaling molecules in many pathophysiological processes. Although sphingolipids comprise a small part of the plasma lipidome, some plasma sphingolipids are recognized as implicated in the development of metabolic diseases and cardiovascular diseases. Plasma sphingolipids are mostly carried out into lipoproteins and may modulate their functional properties. Lipids ingested from the diet contribute to the plasma lipid pool besides lipids produced by the liver and released from the adipose tissue. Depending on their source, quality and quantity, dietary lipids may modulate sphingolipids both in plasma and lipoproteins. A few human dietary intervention studies investigated the impact of dietary lipids on circulating sphingolipids and lipid-related cardiovascular risk markers. On the one hand, dietary saturated fatty acids, mainly palmitic acid, may increase ceramide concentrations in plasma, triglyceride-rich lipoproteins and HDL. On the other hand, milk polar lipids may decrease some molecular species of sphingomyelins and ceramides in plasma and intestine-derived chylomicrons. Altogether, different dietary fatty acids and lipid species can modulate circulating sphingolipids vehicled by postprandial lipoproteins, which should be part of future nutritional strategies for prevention of cardiovascular diseases.

## 1. Introduction

There is a large consensus for the implication of dietary fats on cardiometabolic health and on nutrition-driven diseases. Beneficial or harmful effects of dietary fats (e.g., butter, margarine, oils, fat spread…) on cardiovascular events partly depend on the specific sources of dietary lipids, their structures and their amounts (1). Triglycerides, representing the major dietary lipids, modulate fasting and postprandial lipemia and modify the lipid composition of triglyceride-rich lipoproteins (TGRL), contributing to the incidence of various cardiovascular events (2). Advances in lipidomic analyses by mass spectrometry have helped to identify minor lipid species as surrogate markers of cardiovascular risk. Among them, sphingolipids constitute a very diverse category of lipids with different classes and more than a thousand individual molecular species (3, 4). Sphingolipids are composed of a long-chain amino-alcohol backbone as a sphingoid base (mostly d18:1 sphingosine in humans) to which a fatty acid is attached by an amide bond. Each sphingolipid class presents numerous molecular species varying in chain length (from C14 to C26), degree of saturation (mostly saturated and monounsaturated) and degree of hydroxylation. In plasma, sphingolipids account for 5% of total lipids. Among them, sphingomyelins (SM) represent 10% of total plasma phospholipids and account for 96% of total plasma sphingolipids while ceramides (Cer) represent 3% of plasma sphingolipids (3). Other plasma sphingolipids are glycosphingolipids including glucosylceramides (glucosylCer) and gangliosides as well as the bioactive sphingosine and sphingosine-1-phosphate molecules. Lipoproteins are the main carriers of plasma sphingolipids. Very few clinical studies have focused on the modifications of surface sphingolipids in lipoproteins, which might potentially alter the functional properties of lipoproteins, especially in cardiometabolic diseases. Although sphingolipids represent a small part of the plasma and lipoprotein lipidome, some of them are important structural and bioactive signaling molecules in mammals, and some have significant roles in the development of metabolic disorders (5). Indeed, specific Cer molecular species in plasma are known as potent predictive biomarkers of cardiovascular disease occurrence (6). Pharmacological treatments and nutritional interventions can modulate circulating sphingolipids in cancer and metabolic diseases respectively, and drugs targeting sphingolipid metabolism have indeed shown some promising results in anti-cancer therapeutics (7). The source, quality and quantity of dietary lipids may also impact circulating sphingolipids and possibly prevent nutrition-driven metabolic diseases. This mini-review will examine the effects of dietary fatty acids and SM on circulating sphingolipids, and will be mainly focused on human studies.

## 2. Dietary fatty acids and circulating sphingolipids

### 2.1. Effects of dietary fatty acids on plasma sphingolipids

Observational studies drew attention to the positive associations between plasma Cer concentrations and incident clinical events of cardiovascular diseases, and to the modulation of plasma sphingolipids by healthy diets. In the prospective case-cohort PREDIMED study (8), plasma concentrations of four Cer molecular species, Cer(d18:1/16:0), Cer(d18:1/22:0), Cer(d18:1/24:0) and Cer(d18:1/24:1) were positively associated with the incidence of cardiovascular events (nonfatal acute myocardial infarction, nonfatal stroke, or cardiovascular death). Moreover, a Mediterranean diet enriched with extra virgin olive oil or nuts might mitigate the potential deleterious effects of elevated plasma Cer concentrations on cardiovascular disease risk in participants initially free from cardiovascular diseases. Similarly, adherence of 93 overweight participants for 12 weeks to an healthy Nordic diet (consisting of whole grains, fruits, vegetables, berries, vegetable oils and margarines, fish, low-fat milk products, and low-fat meat) resulted in decreased plasma concentrations of Cer and especially Cer(d18:1/22:0), Cer(d18:1/23:0) and Cer(d18:1/24:0) when compared to participants assigned to a control Nordic diet (including low-fiber cereal products, dairy fat-based spreads, regular-fat milk products, and a limited amount of fruits, vegetables, and berries) (9). Fasting plasma Cer concentrations correlated positively with total cholesterol and LDL cholesterol concentrations, which is in favor of beneficial effects on cardiovascular risk by modulating Cer levels with healthy dietary habits.

Recently, data from the Epidemiological Study on the Insulin Resistance Syndrome (D.E.S.I.R.) cohort highlighted the relationships between the consumption of dairy products and plasma sphingolipidomic profile. In 105 women from this prospective French cohort, higher consumption of fresh dairy foods, except cheese, was associated with lower plasma total concentrations of dihydroceramides (dihydroCer), direct precursors of ceramides, and a trend toward lower plasma Cer concentrations (10).

Based on cross-sectional data, a large scale sphingolipidomic analysis of plasma from 2,860 ethnic Chinese Singaporeans showed positive associations between the intake of dietary saturated fatty acids (SFA) estimated from food frequency questionnaires and higher plasma concentrations of sphingolipids with a d16:1 sphingoid base backbone, including Cer, monohexosylceramides, dihexosylceramides, SM and sphingosine 1-phosphate (11). Higher intake of polyunsatured fatty acids (PUFA) was associated with lower plasma long-chain Cer and long-chain hexosylceramides concentrations, while monounsaturated fatty acid intake was not associated with plasma sphingolipid concentrations (11).

More targeted studies on the specific effects of fatty acids that constitute dietary lipids showed differential effects of fatty acids on circulating sphingolipids depending on their saturation or unsaturation and their fatty acid family, i.e. ω3 or ω6 or ω9. A high-fat diet, enriched in SFA, resulted in the accumulation of Cer in plasma, liver and muscles, and was associated with adverse cardiovascular events (12, 13). In 14 overweight subjects, a 1,000 kCal/day overfeeding during 3 weeks with a diet including SFA (30 g coconut oil, 40 g butter and 100 g blue cheese 40% fat per day) resulted in increased plasma total Cer concentrations and particularly long-chain Cer(d18:1/24:0), Cer(d18:1/24:1) and dihydroceramide Cer(d18:0/24:0) as well as hepatic TG concentrations (14). No change in plasma Cer concentrations was observed in 12 subjects consuming a hypercaloric diet comprised of unsaturated fatty acids (14). Similarly, another double-blind randomized nutritional intervention study performed in 61 middle-aged overweight subjects for 8 weeks showed differential effects of diets enriched with different oils on some serum sphingolipids (15) when relative changes from baseline to post-intervention were compared between the two diets. Overfeeding with muffins containing palm oil, rich in palmitic acid (C16:0), resulted in increased serum Cer, dihydroCer and glucosylCer concentrations, while overfeeding with muffins containing sunflower oil, rich in linoleic acid (C18:2 ω6), decreased serum dihydroCer, glucosylCer and lactosylceramides (15). It was hypothesized that an increased intake of palmitic acid and SFA may have stimulated the *de novo* synthesis of Cer in the liver *via* the condensation of palmitoyl-CoA and serine by serine-palmitoyltransferase. These results are in line with studies in rodents showing that the dietary fatty acid composition impacts the main pathways of Cer generation *via* SM hydrolysis and *de novo* synthesis, and degradation by regulating ceramidase activities (16).

Regarding the effects of long-chain ω3 PUFA, eicosapentaenoic acid (EPA, C20:5 ω3) and docosahexaenoic acid (DHA, C22:6 ω3), several randomized controlled trials demonstrated their health benefits for secondary prevention of cardiovascular diseases, although still controversial (17, 18). In particular, high doses (>2 g/day) of ω3 PUFA lowered plasma TG concentrations (19) resulting primarily from the decrease in hepatic VLDL-TG production, and secondarily from the increase in VLDL clearance (20). However, very few data in humans are available on the effects of ω3 PUFA on circulating polar lipids. Rodent studies reported that EPA and DHA may reduce Cer concentrations in muscles, liver and adipose tissue (21, 22). In 33 patients with recent acute myocardial infarction or unstable ischemic attack, an 8-week consumption of fatty fish at least 4 times a week led to decreased concentrations of plasma Cer, diacylglycerols and lysophosphatidylcholines (23), in addition to increased proportions of plasma EPA, docosapentaenoic acid (C22:5 ω3) and DHA as expected. It remains to be determined whether lower plasma Cer concentrations might be part of the cardioprotective effects of long-chain ω3 PUFA.

### 2.2. Effects of dietary fatty acids on sphingolipids in lipoproteins

Sphingolipids are mostly transported in the bloodstream within lipoproteins (5) and are present at different amounts in the lipoprotein classes: VLDL, LDL and HDL (4, 24–26). To get a deeper insight into the dietary lipid-induced modifications of plasma sphingolipids, it is of key importance to determine the concentrations of sphingolipid classes into intestine-derived lipoproteins (27, 28). The intestine produces triglyceride-rich lipoproteins (TGRL), including chylomicrons, apoB48-containing VLDL (29), but also apoA-I, the principal apolipoprotein of HDL (30, 31). A randomized parallel group study in 30 obese type 2 diabetic patients compared the effects of a single fat meal on postprandial TGRL collected 4 h after ingestion of 20 g lipids from a hazelnut-cocoa palm oil-rich spread or butter (28); among fatty acids in each fat source, palmitic acid represented 33 mol% and 42 mol% of total fatty acids respectively. Following the consumption of either meal rich in SFA, total Cer concentrations were higher in postprandial TGRL compared to fasting TGRL, regardless of the saturated fats consumed. Detailed analyses of the sphingolipid molecular species enabled to show specific increases of Cer(d18:1/16:0) and very long chain Cer(d18:1/24:0), Cer(d18:1/24:1). No modification of SM and ganglioside GM3 concentrations nor sphingoid base concentrations were observed between postprandial TGRL and fasting TGRL, nor between the two breakfasts. These results confirm in humans that the supply of dietary palmitic acid may increase *de novo* synthesis of Cer resulting in increased concentrations of Cer in postprandial TGRL. This clinical study also shows that the ingestion of a single fat-rich meal from a plant-based or dairy source by type 2 diabetic patients is sufficient to modify the sphingolipid profile of postprandial TGRL, and potentially their functional properties. Complementary results of the same study indeed showed that postprandial TGRL induced acute *in vitro* platelet activation, *via* increased agonist-induced platelet aggregation and synthesis of thromboxane A_2_ (32), which may contribute to the increased atherogenicity of postprandial TGRL in type 2 diabetes (2). Extending the study with a longer duration and higher amounts of lipids could have led to stronger modifications of the sphingolipidomic profile of lipoproteins. During the postprandial state and hydrolysis of TG from TGRL by lipoprotein lipase, a rapid remodeling of TGRL occurs. Surface lipids of TGRL, including phospholipids and SM, are transferred to HDL *via* the phospholipid transfer protein (5). The intestine also synthesizes some HDL particles. It is then conceivable that the SFA-induced increase of TGRL Cer in turn may have effects on the sphingolipidome of HDL. One study investigated the effect of a high-fat meal composed of heavy whipping cream on fasting and postprandial HDL in 16 healthy subjects (33). The consumption of this SFA-rich meal led to increases in Cer(d18:1/22:0), Cer(d18:1/24:0), Cer(d18:2/24:0), SM(d18:1/14:0), and lactosylceramide LacCer(d18:1/16:0), as well as some phosphatidylcholine molecular species. Since HDL phosphosphingolipidome may strongly affect the atheroprotective functionalities of HDL (34), it can be speculated that SFA-induced higher Cer in postprandial HDL might have led to altered functionalities of HDL.

The metabolism of lipoproteins is highly dynamic and thereby all lipoproteins classes, including liver-derived VLDL and LDL, can be modified by exogenous dietary lipids. In this regard, a pilot study in 6 hypertriglyceridemic men showed that the supplementation for 8 weeks with 3 g/d fish oil rich in EPA and DHA decreased fasting TG in plasma and large VLDL but also Cer concentrations in VLDL, without any modification of SM concentrations in VLDL (35). Regarding LDL, a dietary intervention study comparing the effects of different types of fats on plasma lipoproteins in 36 overweight middle-aged subjects showed that a 3-week consumption of extra daily 1,000 kCal coming from SFA resulted in modifications of LDL composition, independently of LDL cholesterol (36). Excess consumption of SFA (30 g coconut oil, 40 g butter, 100 g blue cheese 40% fat), but not unsaturated fats (36 g olive oil, 266 g pesto, 54 g pecan nuts, 20 g butter), increased concentrations of SM, Cer and saturated TG in LDL and increased their susceptibility to aggregate and consequently their atherogenicity. Altogether, these studies show that the type of fatty acids in the diet affects the sphingolipidomic composition of plasma lipoproteins and consequently their functional properties and lipid-related cardiovascular risk markers.

## 3. Dietary sphingolipids and circulating sphingolipids

### 3.1. Absorption and transport of sphingolipids

Dietary sphingolipids are a minor part of dietary lipids compared to triglycerides. Their intake has been estimated to be around 300–400 mg/day in the United States (37). In Japan, total amounts of sphingolipids, i.e., SM and glucosylCer, in a typical 3,000 kCal meal represent 128–292 mg (38). Dairy products are the primary sources of sphingolipids in the diet, followed by meats, eggs, fishes and some vegetables (37). Polar lipids are the main constituents of the milk fat globule membrane and mainly include 21–24% SM, 25–36% phosphatidylethanolamine, 27–29% phosphatidylcholine, 8–12% phosphatidylserine and 8% phosphatidylinositol (39, 40). Pioneering work from Nilsson group showed that SM are not absorbed intact (41). In the small intestine, SM are sequentially hydrolyzed by alkaline sphingomyelinase to phosphocholines and Cer, which are hydrolyzed to sphingosine and unesterified fatty acids by a neutral ceramidase. The mechanism of incorporation of sphingolipids derived from dietary SM into lipoproteins remains not precisely known. Studies in lymph duct cannulated rats fed with a triolein emulsion together with radiolabeled SM and Cer showed that traces of intact SM or Cer were incorporated into chyle lipids. Regarding absorption of free sphingosine, a metabolite of SM, <10% tritiated sphingosine was recovered into chyle lipids, mainly into TG and fatty acids and a small percentage into Cer and SM (42).

### 3.2. Effects of dietary sphingolipids on circulating lipids

Several initial studies in rodents showed benefits of milk polar lipids and SM supplementations on plasma cholesterol levels [reviewed by Norris et al. (43)] *via* a SM-induced inhibition of cholesterol absorption. A few studies in humans determined the effects of diets enriched in dairy products on plasma lipids. A 4-week supplementation of 48 healthy volunteers with a buttermilk-based formulation enriched with sphingolipids (700 mg SM, 180 mg glucosylCer and 95 mg gangliosides) had no effect on fasting plasma TG, cholesterol, LDL-cholesterol and HDL-cholesterol concentrations (44). By contrast, a 4-week supplementation with 45 g/day buttermilk composed of 187 mg total phospholipids and 24 mg SM reduced serum concentrations of TG (-11%) and total cholesterol (-3%) in 34 healthy volunteers with fasting LDL-cholesterol concentrations of 3.75 mM (1.45 g/L) (45). Besides fasting TG, postprandial TG are independently associated with the incidence of cardiovascular events (46). Therefore, the influence of dietary lipids on the postprandial plasma sphingolipidome needs to be determined. In 18 healthy volunteers, a high fat (40 g) standard breakfast including 975 mg sphingolipids had no effect on postprandial plasma lipid levels measured from 1 to 7 h after the meal consumption, compared to a placebo drink of skim milk powder (47). In 16 normolipemic obese men, the consumption for 4–6 weeks of a breakfast comprising 54 g lipids issued from dairy fat or soybean oil modified the postprandial plasma phospholipid concentrations in relation with the source of dietary fat (12). The concentrations of dihydroCer, SM, GM3 ganglioside, dihexosylceramide, plasmalogens, phosphatidylcholine and phosphatidylinositol increased 4 h after the dairy breakfast compared to fasting concentrations. Despite similar lipemic responses and increased TG concentrations compared to the dairy meal, the consumption of the soy meal did not result in any changes of plasma phospholipids but decreased SM and ganglioside GM3 concentrations 1 h after the soy meal.

To go further on the effects of milk polar lipids on markers of cardiometabolic risk, the next step is to determine their effects in patients at risk for metabolic and cardiovascular diseases and to consider the molecular species of sphingolipid classes. Only one comprehensive dietary intervention study in overweight post-menopausal women at risk of cardiovascular diseases explored the impact of milk polar lipids on some lipid cardiovascular risk markers and also on the circulating concentrations of SM and Cer in plasma and chylomicrons both at fasting and during the postprandial state (48). Milk polar lipids were provided by a cream cheese enriched with 5 g polar lipids *via* a butterserum concentrate. The 4-week daily supplementation with milk polar lipids led to decreased serum TG (-15%), LDL-cholesterol (-9%), and increased HDL-cholesterol (+5%) concentrations as well as to decreased postprandial concentrations of TG and cholesterol in intestine-derived chylomicrons in volunteers compared to volunteers who daily consumed cream cheese devoid of polar lipids (containing only milk TG). In addition, circulating concentrations of sphingolipids were modified by the consumption of milk polar lipids (27). In fasting serum, the second most abundant Cer species, Cer(d18:1/24:1) as well as SM(d18:1/16:1), SM(d18:1/18:1), SM(d18:1/20:1) decreased in the group who consumed 5 g/day of milk polar lipids while serum concentrations of total SM, Cer and phospholipids did not differ significantly between groups. In intestine-derived chylomicrons from the milk polar lipid group, total SM concentrations decreased and total Cer concentrations tended to decrease after the 4-week intervention vs. before intervention. Regarding the molecular species within each class of SM and Cer in chylomicrons, several molecular species of SM relative to CM TG decreased after milk polar lipid intervention, i.e., SM(d18:1/16:0), SM(d18:1/18:0), SM(d18:1/16:1), SM(d18:1/18:1), SM(d18:1/20:1), SM(d18:1/24:0), SM(d18:1/24:1), as well as very long chain Cer species Cer(d18:1/22:0), Cer(d18:1/24:0) and Cer(d18:1/24:1). These observed decreases of SM and Cer molecular species within chylomicrons after milk polar lipid intervention might be due to the endogenous metabolism of sphingolipids inside the enterocytes following the supply of exogenous dietary SM and the supply of one of its degradation products such as sphingosine and/or Cer (41).

## 4. Conclusion and perspectives

**Figure 1:**
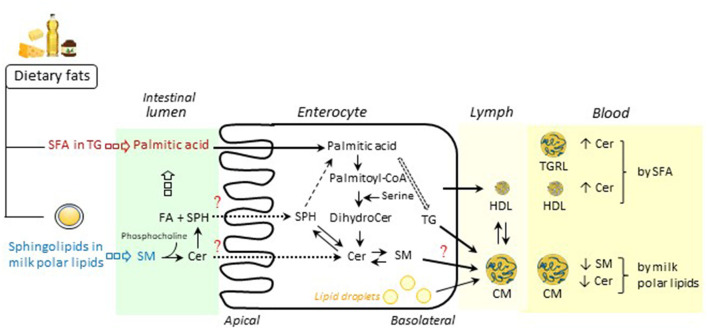
Schematic overview of postprandial intestinal metabolism of dietary lipids, especially sphingolipids. Following ingestion of dietary fats, triglycerides (TG) including saturated fatty acids (SFA) are digested in the intestinal lumen by pancreatic lipases into 2-monoacylglycerols and unesterified fatty acids (FA) (e.g., palmitic acid), which are then absorbed in the enterocytes, resynthesized as TG and packaged into chylomicrons (CM) for secretion into lymph. In the enterocyte, condensation of palmitoyl-CoA and serine by serine palmitoyltransferase (SPT) produces dihydroceramide (dihydroCer) and Cer. Among milk polar lipids including sphingolipids, sphingomyelin (SM) is hydrolyzed by an alkaline sphingomyelinase to ceramide (Cer) and phosphocholine. Cer is further hydrolyzed by a neutral ceramidase into sphingosine (SPH) and FA. In the enterocyte, SPH is mainly converted to palmitic acid, which is incorporated into TG but may be reincorporated into Cer and complex sphingolipids. Intestine-derived lipoproteins, i.e., CM, the major triglyceride-rich lipoproteins (TGRL) and HDL, are released into the bloodstream *via* the lymph. As described in the present review, SFA consumption may lead to increased concentrations of Cer in TGRL (28) and HDL (33). Supplementation with milk polar lipids may result in decreased concentrations of SM and Cer in CM (27). Future research should study the endogenous metabolism of sphingolipid species in enterocytes. Some figure elements were obtained from Servier Medical Art (https://smart.servier.com/).

Circulating sphingolipids can be modulated by exogenous dietary lipids (Figure 1). Observational studies and nutritional intervention trials showed that dietary palmitic acid as well as milk polar lipids and dietary SM modified the concentrations of some sphingolipids in plasma but also in lipoprotein classes (summarized in Table 1). In particular, some specific molecular species of Cer and SM, rather than total Cer and SM, were modified in postprandial TGRL and HDL, which could modify the functionalities of lipoproteins, especially in the context of nutrition-driven chronic diseases. Detailed analyses of sphingolipid classes and molecular species in intestine-derived lipoproteins and also in liver-derived ones could unravel the mechanisms that link sphingolipids species modified by dietary lipids and cardiovascular risk outcomes. It will help to identify novel sphingolipid molecular species in lipoproteins as biomarkers of cardiovascular disease risk, independently of LDL-cholesterol and TG (49). Most clinical data indicate that circulating Cer are modified by dietary SFA and SM. Considering that Cer are at the center of sphingolipid metabolism, it is likely that dietary lipids may also modify the concentrations of other sphingolipid derivatives including sphingoid bases. Therefore, the endogenous metabolism of sphingolipids, especially Cer and sphingosine, inside enterocytes has to be fully elucidated.

**Table 1:** Impact of dietary fatty acids and sphingomyelin on circulating sphingolipids in the randomized controlled trials mentioned in the text.

**Participants**	**Dietary intervention and duration**	**Main fat sources**	**Effect on circulating sphingolipids**	**References**
38 overweight subjects	3-week 1,000 kcal/day overfeeding with SFA or PUFA	SFA: 30 g coconut oil; 40 g butter; 100 g blue cheese 40%fat PUFA: 36 g olive oil; 26 g pesto; 54 g pecan nuts; 20 g butter	SFA: ↑ [Cer] in plasma, especially Cer(d18:1/24:0), Cer(d18:1/24:1) and Cer(d18:0/24:0). ↑ [Cer] and [SM] in LDL PUFA: no change in plasma and LDL sphingolipids	(14, 36)
61 overweight subjects	8-week overfeeding with SFA or PUFA	SFA: palm oil muffins PUFA: sunflower oil muffins	SFA: ↑ [Cer], [dihydroCer], [glucosylCer] in serum PUFA: ↓ [dihydroCer], [glucosylCer] and [lactosylCer]	(15)
33 patients with myocardial infarction or unstable ischemic attack	8-week intake of 4 fatty fish meals per week	100–150 g salmon, rainbow trout, baltic herring, whitefish per meal.	↓ [Cer] in plasma	(23)
30 type 2 diabetic women	SFA-rich breakfast	20 g lipids from hazelnut-cocoa palm oil-rich spread or butter	↑ [Cer] in postprandial TGRL, especially Cer(d18:1/16:0), Cer(d18:1/24:0) and Cer(d18:1/24:1)	(28)
16 healthy subjects	SFA-rich meal	104 g heavy whipping cream	↑ Cer(d18:1/22:0), Cer(d18:1/24:0), Cer(d18:2/24:0), C14:0-SM and lactosylCer in postprandial HDL	(33)
6 men with hypertriglyceridemia	8-week supplementation with 1 g fish oil 3 times a day	Fish oil: 60 mg EPA and 240 mg DHA	↓ [Cer] in VLDL. No change of [SM] in VLDL	(35)
16 obese men	4–6-week consumption of a dairy-based or soy oil-based meal	Dairy meal: 54 g lipids from cheddar cheese (60 g), butter (20 g) and whole milk (300 mL) Soy meal: 50 g lipids from soy cheese (100 g) and soy beverage (300 mL)	Dairy meal: ↑ [dihydroCer], [SM], [GM3], [dihexosylCer] in postprandial plasma compared to fasting plasma Soy meal: ↓ [SM] and ↓ [GM3] in postprandial plasma	(12)
58 postmenopausal women	4-week supplementation with milk polar lipids or cream cheese devoid of polar lipids	Cream cheese enriched with 5g polar lipids from butterserum	Milk polar lipids: ↓ Cer(d18:1/24:1), ↓ SM(d18:1/16:1), SM(d18:1/18:1), SM(d18:1/20:1) in serum ↓ [SM] and ↓ Cer(d18:1/24:1) in chylomicrons	(27)

Finally, there is a need for additional randomized controlled trials in healthy subjects and patients at increased cardiovascular risk to support the beneficial impact of dairy products including polar lipids on circulating lipids, including sphingolipids, lipid metabolism and cardiometabolic health.

## Author contributions

CC wrote the original draft of the document. CV, AP, DC, and M-CM critically revised the document. All authors have made a substantial and intellectual contribution to the work and approved the final version of the manuscript.
